# Reported 1-year prevalence of occupational musculoskeletal disorders in Ontario chiropractors

**DOI:** 10.1186/s12998-020-00345-2

**Published:** 2020-10-23

**Authors:** Samuel J. Howarth, Anser Abbas, Sheilah Hogg-Johnson, Silvano Mior

**Affiliations:** 1 Division of Research and Innovation, Canadian Memorial Chiropractic College, Toronto, ON Canada; 2 Human Performance Research, McMorland Family Research Chair in Mechanobiology, Canadian Memorial Chiropractic College, Toronto, ON M2H 3J1 Canada

**Keywords:** Chiropractic, Standardised Nordic musculoskeletal questionnaire, Manual therapy, Occupational injuries, Workplace health and safety

## Abstract

**Background:**

Chiropractors are a particular subset of health care professionals that reportedly suffer occupational musculoskeletal disorders (MSDs), yet they have received minimal attention to date regarding mitigating risks of occupational injury. Our study determined the prevalence of occupationally-related MSDs in the preceding year, their bodily distribution, severity, and practice-related changes in practicing chiropractors in the province of Ontario.

**Methods:**

We conducted a cross-sectional survey of chiropractors who were members of the Ontario Chiropractic Association (OCA) from January to March 2019. A three-part online survey was developed to ask chiropractors about specific details of MSDs they experienced in the past year and any practice-related changes they made as a result. Responses from participants provided both quantitative and qualitative data. Prevalence estimates were derived for quantitative data. Qualitative data were stratified by themes that were further divided into categories and subcategories. Demographic variables of the respondents and OCA membership were compared to determine representativeness.

**Results:**

From the 432 responses (11.8% response rate), 59.1% reported experiencing an occupationally-related MSD in the past year. Survey respondents were demographically representative of the OCA membership. MSDs were most commonly reported for the lower back (38.3%), wrists/hands (38.1%) and neck (37.4%). Positioning/performing manipulation was the most common occupational activity for MSD of the upper extremity (53.1%) and lower back (34.8%). Chiropractors largely reported their MSDs did not prevent them from doing their normal work (77.4%), despite the fact that 43.2% reported experiencing their MSDs for more than 30 days in the previous year. Common reported work modifications were grouped under themes of practice and physical changes. Practice changes included reducing patient volume, hiring personnel and scheduling. Physical changes included using different office equipment, selecting different techniques requiring lower force and altering their hand contacts or body position when treating patients.

**Conclusions:**

One-year prevalence of occupational MSDs from this study are comparable to previously reported estimates in chiropractors. These data suggest that chiropractors continue with their regular workload despite their MSDs, thereby increasing their chances of presenteeism. Chiropractors changing technique or technique parameters due to their MSDs provides direction for future research to reduce exposure to occupational MSD risk factors.

## Background

Private health care workers in the United States continue to have an incidence rate for occupational injuries and illnesses that is comparable to workers in other physically demanding occupations, such as construction and manufacturing, and account for the highest total number of annual cases [1]. This persistent trend has stimulated substantial research efforts, and prioritization by public funding agencies, to understand musculoskeletal disorder (MSD) risk factors and develop ergonomic solutions to reduce injury. The health care workers studied include nurses, dental professionals, paramedics, and allied health professionals [2–4]. Chiropractors are a particular subset of health care professionals that reportedly suffer occupational MSDs [5–9]; however, to date they have received minimal attention regarding mitigating risks of occupational injury. A preliminary step toward effectively mitigating occupational MSD risks is to understand the nature and body areas commonly injured in chiropractors, the occupational duties perceived to be the source of these injuries, and the impact they have on the personal and professional lives of chiropractors.

Mior and Diakow [6] published the first findings of MSD prevalence in Canadian chiropractors, focussing primarily on back pain. They reported that the lifetime prevalence of back pain amongst chiropractors was 87, and 74% specifically for lower back pain. Subsequent surveys have broadly focused on prevalence of MSDs in American (prevalence estimates of 40.1 and 57%) and South African (prevalence estimate of 69%) chiropractors over their careers [7–9]. A recent survey of Danish chiropractors reported a prevalence of 60.8% for any MSD in the previous year [5]. Collectively, these studies indicate that chiropractors most commonly report MSDs in their upper extremities (thumb, hand, wrist, shoulder) and lower back. The nature of a chiropractor’s work-related MSDs are most frequently attributed to cumulative exposure and overexertion (i.e. sprain/strain types of injuries) [7, 9]. Administering spinal manipulative therapy to patients is consistently reported as the most frequent activity being performed when the MSD was identified [7–9]. Treating patients in a side-lying posture has been reported as particularly problematic for the shoulder and lower back, whereas hand/wrist MSDs are most frequently attributed to performing trigger point therapy [5, 9].

Despite the high prevalence of occupational MSDs in chiropractors, previous surveys have reported that only between 21 to 30% of chiropractors with musculoskeletal complaints have taken time away from work as a result of their MSDs [5, 7, 9]. To cope with their MSDs, chiropractors reportedly modify physical aspects of their treatment delivery such as positioning of their body or the patient’s body, use different techniques (particularly those requiring less force) and change their contact locations. Hansen and colleagues also reported that a third of respondents reduced their working hours as a result of their MSDs [5]. Interestingly, previous studies have typically not reported on other practice-related changes that chiropractors make in response to their MSDs. A more detailed account of specific practice- and technique-related changes made by chiropractors in response to their MSDs is critical for evaluating their impact and developing approaches to mitigate physical risk factors. Furthermore, little is known of the severity of the impact these occupational MSDs have on work and leisure habits of chiropractors.

The aforementioned findings suggest that chiropractors sustain occupational MSDs consistent with overexertion and/or cumulative trauma mechanisms. Since the most recent survey of Canadian chiropractors was conducted over 30-years ago, our primary objective was to investigate the prevalence of occupational MSDs in the previous 12 months (i.e. year) amongst practicing chiropractors in the province of Ontario. Secondly, we sought to evaluate the bodily distribution, and severity (both occupationally and leisurely) of these work-related MSDs. Previous evidence also suggests that injured chiropractors modify their techniques for administering spinal manipulative therapy to accommodate their MSDs; however, no information exists that describes either the specific nature of these modifications or other practice-related changes. A final objective was to identify specific technique modifications and practice-related changes made by practicing chiropractors in response to their MSDs.

## Methods

### Participants & recruitment

**Fig. 1:**
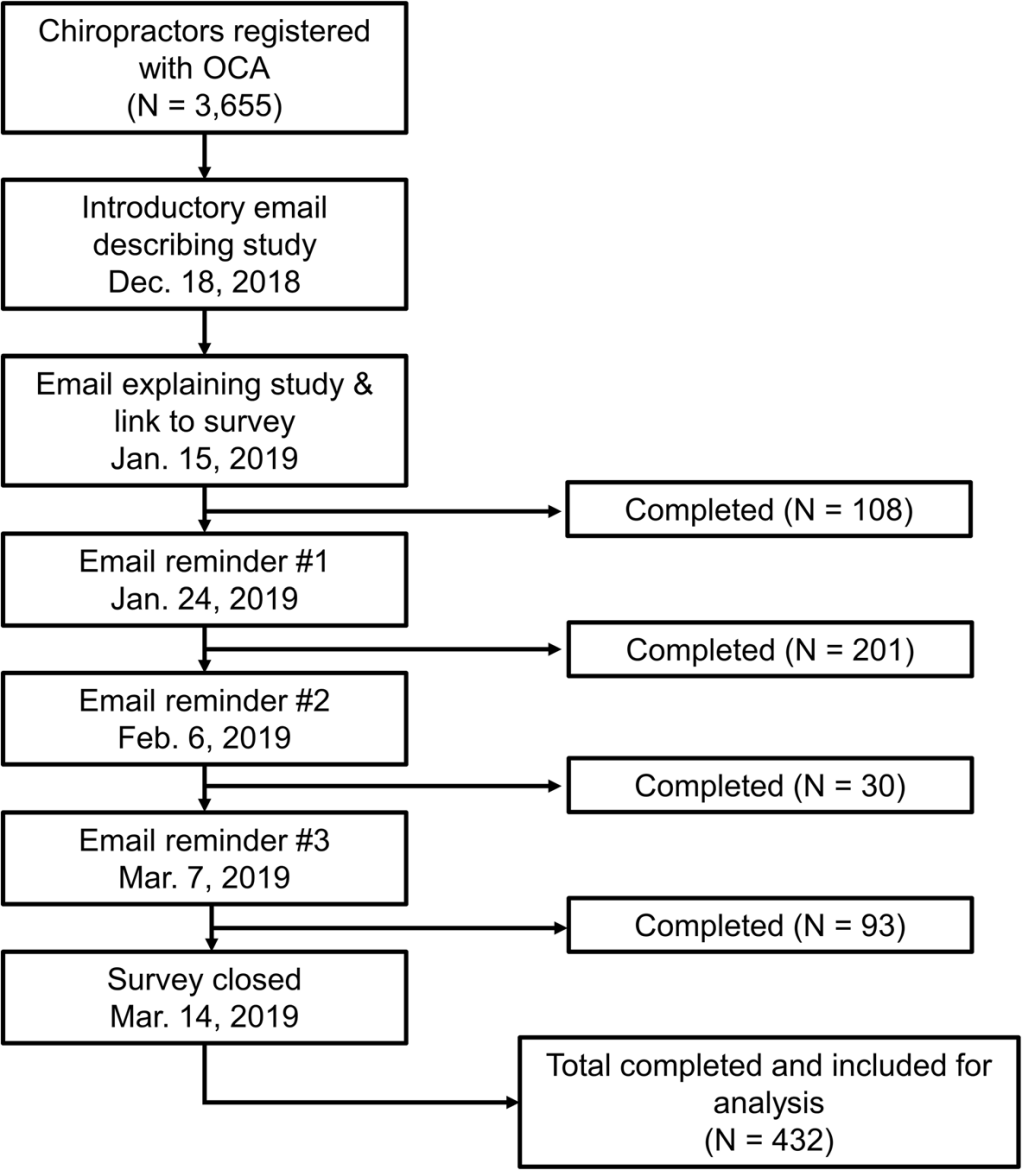
Timeline of notifications and participant recruitment

Chiropractors who were members of the Ontario Chiropractic Association (OCA) between January to March 2019 were invited to participate in the study. We used a modified tailored design method to approach practitioners and optimize participation [10]. This approach included first raising awareness of the study through pre-invitation notices (e.g. articles and advertisements in OCA newsletters), followed by a series of reminder emails inviting individuals to participate in the study by completing an online survey (Fig. 1). In the event non-responding members deleted the initial email with the survey link, subsequent reminder emails contained the same information and link to the survey.

To ensure participant privacy and anonymity, all recruitment materials were electronically distributed by the OCA. Upon opening the survey link, participants were presented with an information letter that outlined details for the study. Participants clicked on the tab at the end of the letter to provide their consent and begin the survey. All procedures and materials for this project were approved by the Research Ethics Board at the Canadian Memorial Chiropractic College (CMCC) prior to beginning recruitment and data collection (REB #1809B01).

### Sample size Goal & Justification

At the time of data collection, there were 3655 registered members of the OCA. Mior and Diakow’s previous study (1987) surveyed chiropractors across Canada and achieved a 60% response rate, but survey burden in general has increased in recent years. Based on the 42.2% response rate from Holm and Rose’s study [9] that issued surveys to 1000 practicing chiropractors in the United States, we expected a response rate of approximately 40% which would yield 1462 responses.

The primary purpose of our survey was to describe the burden of work-related musculoskeletal injury/symptoms among practicing chiropractors, which was expressed as prevalence (with 95% confidence interval) or the proportion of respondents that reported injuries and/or symptoms related to their work as a chiropractor. With a sample size of *N* = 1462, the confidence interval (CI) width for a prevalence estimate of 50% would be +/− 1.3%.

### Survey instrument

**Fig. 2:**
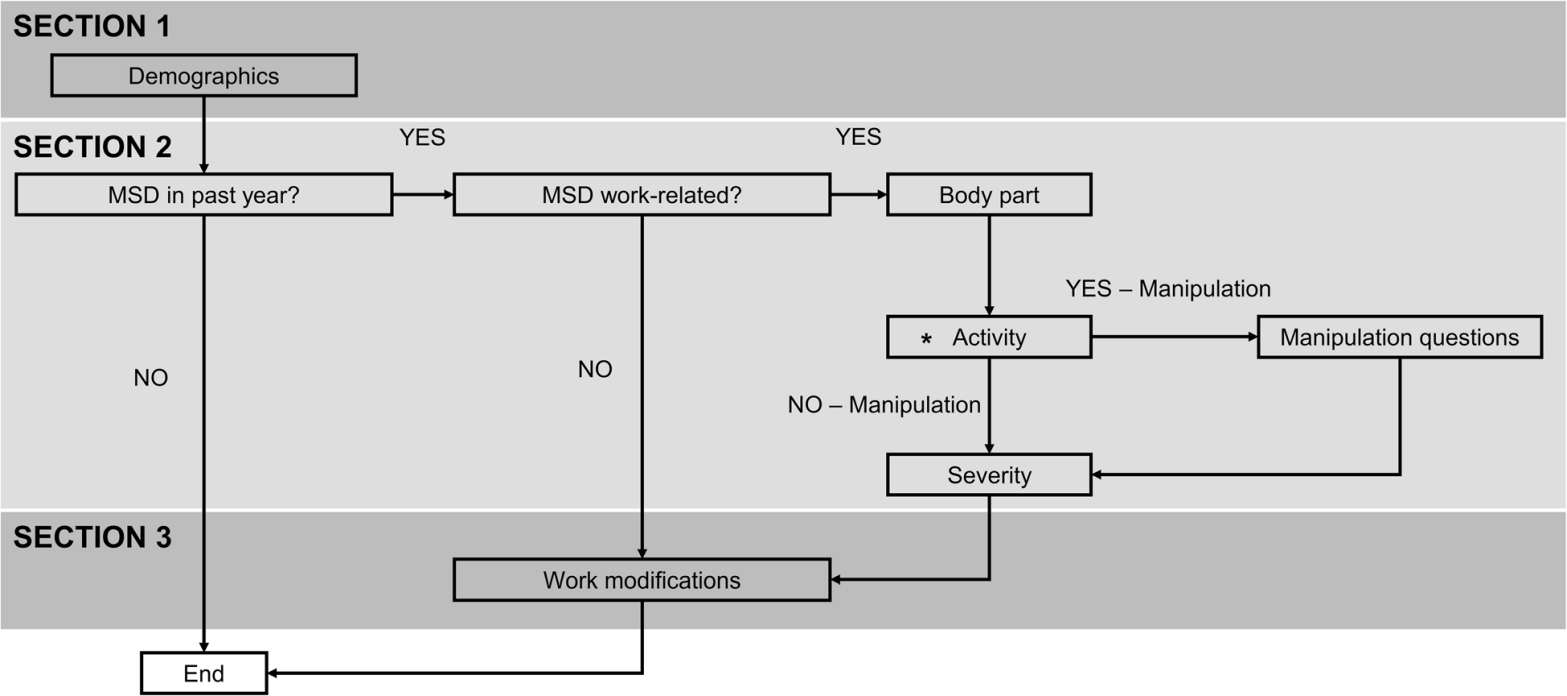
Schematic of survey illustrating three sections and skip logic that was implemented. The asterisk indicates that these sections were repeated for each body part that a respondent reported experiencing work-related musculoskeletal trouble in the previous year

Each consenting participant anonymously completed a custom-designed online survey that was implemented in SurveyMonkey (Ottawa, ON, Canada). The online survey included both closed-ended and open-ended questions and combined elements from the survey used by Holm and Rose [9] and the Standardized Nordic Musculoskeletal Questionnaire (SNMQ) [11] [see Additional file 1]. The SNMQ is a commonly used questionnaire, demonstrated to provide reliable information regarding the onset, prevalence and severity of musculoskeletal pain [11]. Our survey was structured in three sections, asking each chiropractor to provide information related to their: (1) demographics; (2) history (if any) of occupational MSDs related to their practice as a chiropractor in the previous year; and (3) specific work modifications made due to any MSD (Fig. 2). We included built-in skip patterns based on participant response to improve efficiency and limit time to completion.

Demographic data were closed-ended questions related to personal characteristics (e.g. gender, age, height, weight) and practice characteristics (e.g. graduating chiropractic college, geographic location of practice identified by postal code, years in practice, practice volume, practice specialties, daily used techniques, and adjunct therapies).

Next, a single question asked participants if they had experienced a MSD related to their practice as a chiropractor in the previous year. Chiropractors who answered “No” were taken to the end of the survey and thanked for their participation (Fig. 2). Those answering “Yes” were asked to provide additional information related to occupational MSD history in specific regions of the body (neck, shoulders, elbows, wrists/hands, upper back, lower back, hips/thighs, knees, ankles/feet) over their career and in the previous year. As in the SNMQ, we included a shaded body diagram to direct participants’ focus on specific body regions when completing the survey. For participants reporting an occupational MSD in their neck, shoulders, lower back, and/or hands/wrists, there was a set of follow-up questions for each region reported. These follow-up questions were derived from the SNMQ and probed the specific type of their injury/symptom, duration, and severity for the given body region.

In the last section of the survey, we asked two open-ended questions on MSD history. The first asked about particular activities to which chiropractors attributed the onset of their condition(s). And the second probed specific practice changes made by chiropractors to accommodate their MSDs (e.g. using different techniques/adjunct therapies, altering hand contacts and/or body posture for a given technique, reducing hours).

### Data analysis

We calculated averages and standard deviations to describe participant demographics (e.g. age, height, weight, years in practice, and practice volume). De-identified demographic data (age, gender, years in practice, geographic location of practice identified by postal code) that is routinely collected by the OCA from their members was used to evaluate non-responder bias. We compared respondents with the target population using histograms of these demographic variables to assess the representativeness of our sample. Composition for each bin in a histogram was expressed as a percentage of either all respondents (our study) or all members (OCA members).

General prevalence of work-related MSDs (i.e. answering “Yes” to the question about having experienced a MSD related to their practice as a chiropractor in the previous year) was quantified as a percentage of all participants and presented with a 95% confidence interval (CI). Prevalence over the previous year for specific areas of the body was also quantified as a percentage of all participants with 95%CI.

Responses to the open-ended questions related to modifications in response to a MSD were reviewed. We employed qualitative content methodology using manifest analysis to describe what participants actually said [12]. Each participant comment was reviewed, decontextualized and recontextualized, creating codes that were then organized into representative subcategories. The subcategories were then grouped into main categories and stratified into representative themes. Selective representative comments were used to highlight particular categories. Comments were independently reviewed and coded by two authors (SM, SH), who subsequently met to discuss and reach consensus on coding and structuration of the final categories and themes. Such triangulation increases validity and trustworthiness of results [12].

## Results

### Participant demographics

A total of 432 responses were received, which equated to a response rate of 11.8%. Demographics of the sample are summarised in Tables 1 and 2. Approximately four out of every five respondents were trained in Canada (Table 1). Most respondents reported working four or 5 days per week. On average, respondents reported working a total of 38 h per week with approximately a 3:1 ratio of patient contact hours to administrative work (Table 1). The greatest proportion of respondents reported using a stationary table, and the average common table height across all respondents was nearly 2 ft off the ground (Table 1).

**Table 1:** Personal and practice-related demographics of respondents. All values are reported as percentages (*N* = 432)

	(%)
Sex	
Male	60.2
No response	0.5
Age	
25–29	10.0
30–34	13.2
35–39	13.4
40–44	13.7
45–49	18.1
50–54	9.0
55–59	6.9
60–64	7.9
65–69	6.3
70+	1.2
No response	0.5
Chiropractic college country	
Canada	80.1
USA	19.0
UK	0.2
Australia	0.7
No response	0
Practice specialization	
Imaging	0.0
Clinical Sciences	1.6
Rehabilitation Sciences	5.6
Sports Sciences	6.5
Orthopedics	0.9
Other	7.6
No response	81.0
Years in practice	
0–1	5.3
2–5	11.8
6–10	14.6
11–20	30.6
21–30	19.2
31–40	14.6
40+	3.2
No response	0.7
Postal Code	
K	13.4
L	34.0
M	20.8
N	22.7
P	8.6
No response	0.5
Days worked per week	
0	1.2
1	0.7
2	2.8
3	11.6
4	25.7
5	45.1
6	11.3
7	1.6
No response	0
Patient contact hours per week (*N* = 426)	
	28.8 (10.0)
Administrative hours per week (*N* = 425)	
	9.1 (7.6)
Tables used	
Stationary	60.6
High-low	23.8
Both	15.0
No response	0.5
Preferred table height (inches) (*N* = 408)	23.3 (7.3)

**Table 2:** Chiropractic techniques and adjunct therapies used by respondents. Multiple responses were permitted by each respondent for commonly used chiropractic techniques and regular adjunct therapies. All values are reported as percentages (*N* = 432)

Common and most frequently used chiropractic techniques		
	Common (%)	Most Frequent (%)
Activator Methods	40.7	4.6
Applied Kinesiology	5.6	1.6
Cranial	6.0	0.2
Diversified	94.9	81.3
Flexion-Distraction	12.3	1.2
Gonstead	5.6	0.2
Logan Basic	3.9	0.2
Nimmo/Receptor Tonus	2.3	0
Palmer Upper Cervical/HIO	1.2	0
SOT	6.7	0
Thompson	27.1	5.3
Other	13.9	4.6
No response	0.2	0
Regular adjunct therapies	(%)	
Traction	32.4	
Massage	31.9	
Active Release Technique	35.0	
Myofascial Release Therapy	60.9	
Mobilization	72.2	
Exercise	82.6	
Acupuncture	39.1	
Ice/Heat Packs	38.2	
Ultrasound	34.5	
Laser	31.0	
Shockwave	12.0	
Interferential Current	41.9	
Other	10.9	
No response	3.9	

Diversified technique (primarily high velocity, low amplitude thrust technique) was commonly used by nearly all respondents, and 81.3% reported this as their single most frequently used technique (Table 2). Activator methods and Thompson technique were the second and third most commonly used technique by respondents (Table 2). The top three reported adjunct therapies used by respondents were exercise, mobilisation and myofascial release (Table 2).

### Non-responder Bias

**Fig. 3:**
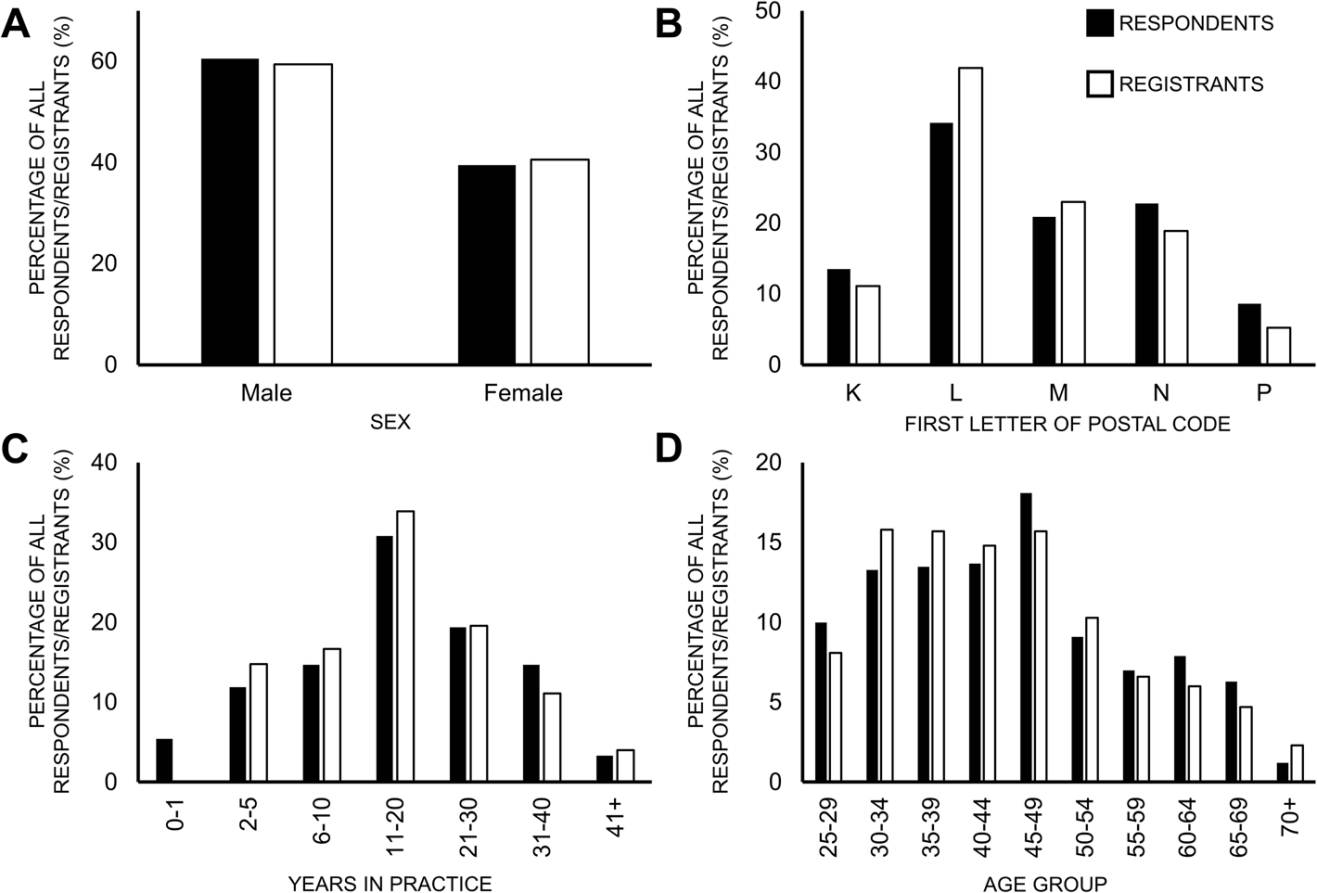
Histograms of sex (**a**), geographic practice location (**b**), years in practice (**c**) and age (**d**) for respondents (black bars) to this survey and registrants (white bars) of the Ontario Chiropractic Association

Histograms graphically demonstrated that our respondents were similar to the target population for the four demographic variables (sex, age, years in practice and geographic location of practice) that were used to assess representativeness (Fig. 3).

### Prevalence and distribution of musculoskeletal disorders

The prevalence of chiropractors experiencing any MSD in the previous year was 91.7% (Table 3). Work-related MSDs, among all respondents (*N* = 428) who provided information related to experiencing any MSD in the previous year, had a prevalence of 59.1% with 95%CI (56.8–61.4%). The three most common areas of work-related MSDs were the lower back, wrists/hands, and neck (Table 3). On average across all body parts, only 21.8% of the reported work-related MSDs experienced in the previous year were a first occurrence; however, this average was affected by a greater percentage of reported upper extremity conditions first occurring within the previous year (Table 4).

**Table 3:** Prevalence of musculoskeletal troubles, and work-relatedness, in past year. Breakdown of work-related musculoskeletal troubles by body part. Each respondent was able to report musculoskeletal troubles for multiple body parts. Respondent ratings of health and recovery expectations from their musculoskeletal troubles. All values are reported as percentages with the corresponding limits for the 95% confidence intervals (CI)

Musculoskeletal trouble last 12 months (N = 432)_	(%)	95%CI
Yes	91.7	(90.9, 92.4)
No response	0.9	
Rating of general health (*N* = 396)	(%)	95%CI
Excellent	26.0	(24.1, 27.9)
Very good	55.8	(53.4, 58.2)
Good	16.7	(15.3, 18.0)
Fair	1.5	(1.4, 1.7)
Poor	0	
No response	0	
Rating of recovery likelihood (N = 396)	(%)	95%CI
Not likely	5.1	(4.6, 5.5)
2	1.8	(1.6, 1.9)
3	1.3	(1.1, 1.4)
4	2.0	(1.8, 2.2)
5	1.8	(1.6, 1.9)
6	5.3	(4.8, 5.8)
7	2.8	(2.5, 3.0)
8	11.6	(10.6, 12.6)
9	10.4	(9.4, 11.3)
Very likely	32.6	(30.4, 34.7)
Already recovered	24.5	(22.7, 26.3)
No response	1.0	
Musculoskeletal trouble work-related? (N = 396)	(%)	95%CI
Yes	63.9	(61.6, 66.2)
No response	0	
Work-related musculoskeletal trouble in last 12 months by body part (N = 428)		
body part	(%)	95%CI
Neck	37.4	(35.2, 39.6)
Shoulders	29.9	(27.9, 31.9)
Elbows	14.5	(13.3, 15.7)
Wrists/Hands	38.1	(35.8, 40.3)
Upper Back	24.3	(22.6, 26.0)
Lower Back	38.3	(36.1, 40.6)
Hips	11.7	(10.7, 12.7)
Knees	11.9	(10.9, 12.9)
Feet/Ankles	8.4	(7.7, 9.1)
No response	1.2

**Table 4:** Activities attributed to work-related musculoskeletal troubles in each body part and whether musculoskeletal troubles were a first-time occurrence. The value of N shown below each body part name represents the denominator used to calculate percentages that are reported in their respective columns

	BODY PART WITH REPORTED MUSCULOSKELETAL TROUBLE								
	Neck	Shoulders	Elbows	Wrists & Hands	Upper Back	Lower Back	Hips	Knees	Feet & Ankles
N	160	128	62	163	104	164	50	51	36
	(%)	(%)	(%)	(%)	(%)	(%)	(%)	(%)	(%)
First occurrence									
Yes	10.6	28.1	40.3	32.5	4.8	12.8	24.0	23.5	19.4
No	86.9	70.3	51.6	62.6	88.5	80.5	70.0	72.5	77.8
No response	2.5	1.6	8.1	4.9	6.7	6.7	6.0	3.9	2.8
Activity									
Diagnostic procedure	0	0.8	0	1.8	0	0	0	2.0	0
Slipping, tripping, or falling	1.3	3.9	3.2	0.6	1.0	1.8	2.0	11.8	8.3
Applying modality	0	0	3.2	1.2	0.0	1.2	0	0	0
Maintaining prolonged position	35.6	6.3	3.2	2.5	39.4	25.6	20.0	15.7	27.8
Lifting	2.5	5.5	9.7	0.6	4.8	8.5	4.0	2.0	0
Demonstrating exercise	0	1.6	0	0.6	1.0	1.8	4.0	7.8	0
Positioning patient for manipulation	7.5	15.6	8.1	4.9	2.9	12.8	2.0	0	5.6
Performing manipulation	20.0	43.0	35.5	52.1	19.2	22.0	18.0	5.9	5.6
Do not remember	14.4	12.5	6.5	6.7	12.5	9.8	16.0	17.6	16.7
Other	17.5	10.2	24.2	25.2	13.5	9.8	28.0	33.3	33.3
No response	1.3	0.8	6.5	3.7	5.8	6.7	6.0	3.9	2.8

The combination of positioning/performing manipulation was the attributable source for 53.1% of work-related MSDs in the upper extremities and 34.8% of those in the lower back (Table 4). Maintaining a prolonged posture was the most frequently attributed source for work-related MSDs in the neck and upper back (Table 4). Work-related MSDs of the trunk (neck, upper back, and lower back) and upper extremities attributed to positioning/performing manipulation were most commonly related to applying Diversified manipulation to the patient’s lower back (Table 5). The greatest proportion of wrist/hand issues and upper back issues attributed to positioning/performing manipulation were experienced when administering treatment to the patient’s upper back (Table 5). Patients were predominately in a side-lying posture for those who reported experiencing symptoms in their neck, shoulders, and lower back while positioning/performing manipulation (Table 5). A more equal division between patient positions of prone, supine and side-lying was identified for MSDs reported by chiropractors in their elbows, wrist/hand and upper back (Table 5).

**Table 5:** Patient position, body part being manipulated, technique used and table height for those who attributed musculoskeletal troubles to performing or positioning a patient for manipulation. The value of N shown below each body part name represents the denominator used to calculate percentages that are reported in their respective columns

	BODY PART WITH REPORTED MUSCULOSKELETAL TROUBLE					
	Neck	Shoulders	Elbows	Wrists & Hands	Upper Back	Lower Back
N	44	75	27	93	23	57
	(%)	(%)	(%)	(%)	(%)	(%)
Part of body manipulating/adjusting						
Neck	4.5	5.3	22.2	18.3	4.3	3.5
Shoulder	6.8	2.7	0	1.1	0	0
Elbow	0	0	0	0	0	0
Wrist/hand	0	0	3.7	1.1	0	0
Upper back	13.6	6.7	7.4	28.0	39.1	3.5
Lower back	65.9	74.7	40.7	33.3	39.1	77.2
Hip	4.5	4.0	3.7	2.2	0	3.5
Knee	0	0	0	0	0	0
Ankle/foot	0	0	0	1.1	0	0
Do not remember	4.5	6.7	22.2	15.1	17.4	12.3
No response	0	0	0	0	0	0
Specific manipulative/adjustment technique used						
Activator methods	0	2.7	7.4	4.3	4.3	1.8
Applied Kinesiology	0	0	0	0	0	0
Cranial	0	0	0	1.1	0	0
Diversified	90.9	96.0	77.8	88.2	91.3	89.5
Flexion-Distraction	0	0	7.4	0	0	1.8
Gonstead	0	0	0	0	0	0
Logan Basic	0	0	0	0	0	0
Nimmo/Receptor Tonus	0	0	3.7	1.1	0	0
Palmer Upper Cervical/HIO	0	0	0	0	0	0
SOT	0	0	0	0	0	0
Thompson	4.5	0	3.7	3.2	4.3	5.3
Do not remember	0	0	0	0	0	1.8
Other	4.5	1.3	0	2.2	0	0
No response	0	0	0	0	0	0
Patient position						
Side-lying	63.6	74.7	37.0	29.0	34.8	71.9
Prone	11.4	8.0	25.9	26.9	30.4	7.0
Supine	20.5	10.7	18.5	29.0	26.1	7.0
Seated	4.5	1.3	3.7	1.1	0	1.8
Standing	0	1.3	3.7	1.1	0	0
Do not remember	0	2.7	11.1	12.9	8.7	8.8
No response	0	1.3	0	0	0	3.5
Approximate table height (inches)						
N	44	74	27	91	23	55
Average	25.3	24.9	23.9	24.8	25.3	23.9
SD	6.9	7.5	4.7	6.2	7.6	6.4

Work-related factors impacting the hips and wrist/hand were most commonly attributed to a chiropractor changing jobs or their occupational duties (Table 6). Generally, respondents with a work-related MSD reported that their condition(s) neither caused them to reduce either their work nor prevented them from doing their normal work (Table 6); however, on average, 43.2% of reported work-related MSDs were experienced for more than 30 days in the previous year. Chiropractors reporting work-related MSDs were most likely to seek treatment if their condition was with respect to the trunk, shoulders or hips.

**Table 6:** Impact and duration of musculoskeletal troubles on personal and professional activities. Only those who reported a total length of time with trouble that was greater than zero days were asked subsequent questions provided in the second portion of the table. The value of N shown below each body part name represents the denominator used to calculate percentages that are reported in their respective columns

	BODY PART WITH REPORTED MUSCULOSKELETAL TROUBLE								
	Neck	Shoulders	Elbows	Wrists & Hands	Upper Back	Lower Back	Hips	Knees	Feet & Ankles
N	160	128	62	163	104	164	50	51	36
	(%)	(%)	(%)	(%)	(%)	(%)	(%)	(%)	(%)
Changed jobs or duties									
Yes	5.6	10.9	8.1	14.7	4.8	11.6	18.0	3.9	2.8
No	91.9	87.5	83.9	80.4	88.5	81.1	76.0	92.2	94.4
No response	2.5	1.6	8.1	4.9	6.7	7.3	6.0	3.9	2.8
Total length of time with trouble									
0 days	0	6.3	4.8	6.1	5.8	7.3	8.0	5.9	8.3
1–7 days	34.4	23.4	21.0	17.2	27.9	26.8	6.0	13.7	11.1
8–30 days	28.1	20.3	25.8	23.3	26.0	31.1	32.0	23.5	25.0
More than 30 days but not every day	28.8	39.1	33.9	35.6	28.8	22.6	26.0	37.3	41.7
Every day	6.9	9.4	6.5	13.5	4.8	5.5	22.0	15.7	11.1
No response	1.9	1.6	8.1	4.3	6.7	6.7	6.0	3.9	2.8
	Neck	Shoulders	Elbows	Wrists & Hands	Upper Back	Lower Back	Hips	Knees	Feet & Ankles
N	157	118	54	146	91	141	43	46	32
	(%)	(%)	(%)	(%)	(%)	(%)	(%)	(%)	(%)
Reduced work activity									
No	93.6	88.1	90.7	85.6	90.1	87.2	79.1	93.5	90.6
Monthly	3.2	1.7	5.6	5.5	1.1	5.0	9.3	2.2	3.1
Weekly	1.9	5.1	1.9	5.5	5.5	6.4	7.0	2.2	6.3
Daily	0	3.4	1.9	3.4	0	0.7	4.7	2.2	0.0
No response	1.3	1.7	0	0	3.3	0.7	0	0	0
Reduced leisure activity									
Yes	22.3	35.6	38.9	30.1	20.9	46.8	67.4	63.0	46.9
No	77.7	62.7	59.3	69.2	78.0	53.2	32.6	37.0	53.1
No response	0	1.7	1.9	0.7	1.1	0	0	0	0
Days normal work prevented									
0 days	88.5	74.6	83.3	73.3	83.5	70.2	62.8	82.6	78.1
1–7 days	8.9	13.6	5.6	11.6	6.6	17.0	11.6	10.9	6.3
8–30 days	1.9	4.2	3.7	6.2	2.2	8.5	14.0	4.3	12.5
More than 30 days	0.6	5.1	7.4	8.9	6.6	4.3	11.6	2.2	3.1
No response	0	2.5	0	0	1.1	0	0	0	0
Sought medical help									
Yes	82.2	66.1	50.0	45.9	84.6	78.0	76.7	43.5	53.1
No	17.8	32.2	50.0	54.1	14.3	22.0	23.3	56.5	46.9
No response	0	1.7	0	0	1.1	0	0	0	0

### Participants perspectives of injuries and preventative strategies

**Fig. 4:**
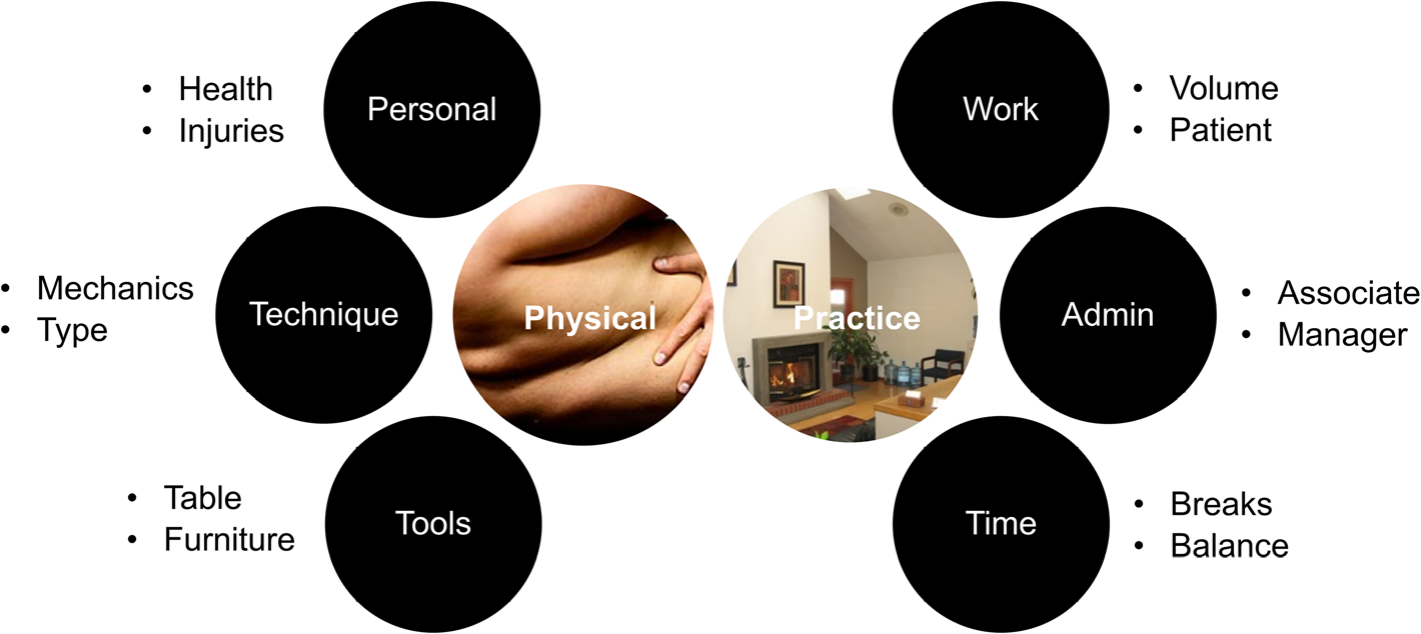
Overview of qualitative findings from open-ended questions related to work modifications that were made as a result of any reported musculoskeletal troubles. Two themes were each stratified by three categories that each had two main subcategories

We explored participants’ opinions about the nature and cause of their MSDs and strategies used to prevent or accommodate their conditions. An overview of these findings is presented in Fig. 4.

Two themes emerged from their responses to our two opened ended questions, physical and practice factors. Physical factors captured attributes categorized as personal, technique specific and office equipment. Participants identified changes to different office equipment they used in the delivery of patient care. In particular, the use of hydraulic technique tables allowing for improved or less stressful body postures, in particular when utilizing different interventions. For example, one participant noted, “*The main change was getting a hydraulic chiropractic table that I can raise up and lower down. This has significantly impacted my lower back health in a positive way!”* (ID#14). Others noted increasingly using table sections that drop or distract, as a means of decreasing the stress and discomfort experienced when using Diversified technique. Participants described the use of mobile stools and ergonomic office equipment to allow for improved posture and limit fatigue. Participants also reflected on personal reasons for making changes, including either work-related or sport-related injuries and physical health problems, such as:

*“Right shoulder is primary concern and I find I’m constantly trying to avoid aggravating it with working positions, especially lumbar side-lying SMT.”* (ID#276) and.

*“I had to ultimately go back to school for another career. I am a small female and my physical problems with chiropractic began almost immediately after finishing chiropractic college.”* (ID#323).

The one physical factor commonly cited by participants related to technique-specific problems and changes. Participants described how they modified their contact, body position or posture to reduce physical stress or forces impacting their body: *“Using a little more care and attention to my own body mechanics and posture.”* (ID 333) Others altered forces used during the delivery of an adjustment/manipulation or soft tissue therapy: *“Less force/pressure when preforming deep tissue massage.”* (ID#1) Whilst others reported changing their technique, relying more on mechanically assisted techniques or other modalities requiring less force than Diversified high-velocity low-amplitude manipulation, as noted in the following quotes:

*“Moved from manual to instrument adjusting.”* (ID#317).

*“Reduced side posture low back adjusting and relied more heavily on activator and drop table techniques.”* (ID#425).

*“Less ART, transitioned to more stretching, traction, Graston, acupuncture.”* (ID#384).

The second emergent theme related to practice factors that addressed strategies related to work, time and office administration. Participants reported making administrative changes to improve office processes and functions. Such changes included hiring associateships, changing office location and having staff assist with work, as noted by a couple of participants:

*“I try to be efficient with my administrative tasks, we’ve hired an office manager to handle the majority of billing issues and I spread my computer time.”* (ID#350) and *“Hired doctor to adjust some patients.”* (ID#362).

Others modified their workday, reducing office hours or introducing more breaks during the day, and scheduling holidays to ensure improved work-life balance. For example, *“We try to schedule our day so we’re not working any longer than 4 hours straight, to have a little “body break”.* (ID#378).

Work-related aspects of practice factors focused not only on patient volume but also patient type. Participants reported focusing on a different patient population as a strategy to overcome their conditions or limiting stresses on their self. For example, a participant noted,

*“Focusing more on pediatrics rather than adult injuries.”* (ID#248),

*“Selected patient population that required less SMT and more mobilization. Self-selected less lower back pain patients.”* (ID#175) and,

*“Reduced 2 full days of practice. Also trying to use more acupuncture to reduce pain and preserve longevity of ability to practice. Trying to aim to market to females and less elite athletes, as I am smaller individual and have less strength now to work on ‘large body types.* (ID#396).

But in response to conditions and dealing with MSD, participants tended to change the volume of patients seen and decrease physical demands resulting from long hours of work, for example, *“Decreased patients seen per day, use more acupuncture techniques than adjustments and activator methods more often.”* (ID#46).

## Discussion

The current study reported on the prevalence of work-related MSDs in practicing chiropractors in the previous one-year period. Our findings indicated that almost 3/5 practicing chiropractors reported a work-related MSD in the previous year with the most commonly affected parts of the body being the lower back, wrists/hands and neck. The combination of preparing the patient for manipulation or performing a manipulation, particularly directed toward the lower back, was the most frequent occupational duty attributed to MSDs of the upper extremities and lower back. Nearly two thirds of respondents claimed to have altered their manipulation technique or technique parameters to accommodate for their MSDs. These prevalence data and specific information on work modification(s) are critical for future targeted investigations evaluating the biomechanics of these modifications and mitigating strategies for reducing the physical risk of MSDs experienced by chiropractors and other health care professionals that use manual therapies.

Data from the current study adds to the growing epidemiology of work-related MSDs reported by chiropractors. Our prevalence estimate for any work-related MSD over the previous year (59.1%) was similar to the estimate provided by a recent study of MSDs in Danish chiropractors (60.8%) [5] and the lifetime prevalence in South African chiropractors (69%) [7]. Interestingly, the two studies that focused on lifetime prevalence of work-related MSDs in American chiropractors reported disparate estimates of 40.1% [9] and 57% [8]. The reason for this difference is unclear. One possible explanation is that Rupert and Ebete [8] restricted their study population to chiropractors with at least 15 years of experience in clinical practice, which may have led to a higher lifetime prevalence estimate. Nonetheless, their prevalence estimate is closer to other estimates obtained over the course of a career and a period of 1 year, as reported in our study. However, although chiropractors may attribute their MSDs to their work, it is unclear if the occupational duties were their actual cause. Our data suggests that most work-related MSDs reported by chiropractors were not a first-time occurrence, which may imply that the occupational physical demands are aggravating symptoms of MSDs.

Our prevalence estimates by body part differ from those reported in previous studies. However, the three main affected areas of the body identified in the current study (lower back, wrist/hand and shoulder) have been consistently ranked among the top three parts of the body that chiropractors report experiencing occupational MSDs [5, 7–9]. In comparison to the only previous study of MSDs in Canadian chiropractors [6], prevalence estimates for the neck and shoulder were higher while estimates for the upper back and lower back were lower in the current study. These comparisons should be interpreted cautiously since Mior and Diakow specified neither the period for their prevalence estimates nor whether reported complaints were work-related [6]. This is particularly relevant to the prevalence estimates that are lower in the current study (i.e. upper back and lower back). Positioning a patient for manipulation or performing manipulation on a patient was commonly reported for upper extremity and lower back conditions. Specifically, shoulder and lower back MSDs were mainly attributed to manipulation with a patient in a side-lying posture, which is consistent with previous work [5, 9]. This is likely due to the requirement for a clinician to impart forces to the patient’s body while adopting an awkward posture of their own.

Biomechanical data that documents postures adopted by, and loads induced on the shoulders and lower back of clinicians performing these maneuvers has yet to be established. These data would be useful in determining physical exposure and mitigation strategies. Interestingly, chiropractors who attributed their hand/wrist MSDs to positioning or performing manipulation were equally divided in terms of the patient’s posture (prone, supine, side-lying). This is somewhat different from data reported by Hansen and colleagues [5] that showed a higher prevalence of wrist MSDs while treating a patient in a prone position. A possible reason for this discrepancy is that our survey only allowed respondents to attribute their MSDs to one patient position, whereas other studies have allowed respondents to attribute their MSDs to multiple sources.

Previous studies have been limited in their reporting of the severity of MSDs in chiropractors. Severity in these studies has often been quantified in terms of time away from work, as well as physical (e.g. technique modification) and practice changes (e.g. reduced hours). Qualitative responses to the open-ended questions in our survey identified themes of physical and practice factors modified by chiropractors as a result of their MSDs. Specific practice factors identified in our study included reduced patient volumes, targeting different patient demographics and hiring an associate or office manager, factors reported in previous studies [5, 8]. Prior research has commonly reported that chiropractors alter their technique (type, contacts, force magnitude) or working posture to accommodate their MSDs [5, 7–9], which are consistent with the physical factors identified in our study. As mentioned, future biomechanical studies would be able to determine loads and joint postures during manipulation with the intent of identifying strategies to reduce acute exposure.

A novel contribution of our study was the application of the SNMQ to assess the yearly duration that chiropractors suffer from work-related MSDs. These data found that nearly 50% of those chiropractors with MSDs of the shoulder or hand/wrist reportedly experienced these troubles for more than 1 month in the previous year. This is particularly troubling for a chiropractor given the extent to which the upper extremities are involved in their delivery of manual care to patients. Yet despite this reported finding, our findings suggest that chiropractors largely did not reduce their working activities as a result of their MSDs. Evidence among nurses suggests that those working with musculoskeletal pain and/or depression report increased errors in patient medications and occurrence of patient falls along with diminished quality of care, as examples of “presenteeism” [13]. Future studies should explore if the quality of patient care delivered by chiropractors who continue to work despite their MSDs is negatively affected.

Our study had limitations. First, the response rate for this survey was only 11.8%. This was lower than the response rates for the five previous studies investigating work-related MSDs in chiropractors (average response rate of 53%) [5–9]. However, our analysis of non-responder bias suggests that our respondents were representative of the entire OCA sample in terms of sex, age, years in practice and geographical location of practice. While this does not tell us that our sample was representative in terms of the prevalence for work-related MSDs in the previous year, it does suggest the respondents were similar to the population as a whole in these demographics. A second limitation was that not all practicing chiropractors in Ontario are members of the OCA. Using a different sampling frame such as the College of Chiropractors of Ontario would have overcome this limitation, since all practicing chiropractors must be licensed with the regulatory College; however, more than 80% of registrants with the regulatory College are also members of the OCA, which suggests that the chosen target population for the current study was likely representative of practicing chiropractors in Ontario. Finally, the list of activities to which a chiropractor could attribute a MSD did not include several other forms of manual therapy that respondents reportedly used on a regular basis (e.g. myofascial release, mobilisation). For example, previous work has reported that chiropractors attribute MSDs of the wrist and thumb to performing trigger point therapy [5]. Future studies should include a more comprehensive list of activities/treatments that chiropractors commonly use in daily clinical practice.

## Conclusions

Our study found the prevalence of work-related MSDs in practicing chiropractors in Ontario was approximately 60% over the previous year, which was consistent with data reported from other parts of the world. Positioning patients for and performing a manipulation were commonly attributed causes of MSDs of the lower back, shoulders and hands/wrists. Future biomechanical work should investigate strategies to mitigate physical risk factors for MSDs in chiropractors such as posture and force.

## Supplementary information

Additional file 1. Survey of MSDs in Ontario chiropractors. Survey instrument used for online implementation. (PDF 56 kb)

## Data Availability

The datasets used and/or analysed during the current study are available from the corresponding author (SH) on reasonable request.

## Abbreviations

MSD(s): Musculoskeletal disorder(s)
OCA: Ontario Chiropractic Association
SNMQ: Standardised Nordic Musculoskeletal Questionnaire

## References

[1] Statistics USBoL. Survey of occupational injuries and illnesses data 2018 [Available from: https://www.bls.gov/iif/soii-data.htm.

[2] Anderson SP, Oakman J (2016). Allied health professionals and work-related musculoskeletal disorders: a systematic review. Saf Health Work.

[3] Ngan K, Drebit S, Siow S, Yu S, Keen D, Alamgir H (2010). Risks and causes of musculoskeletal injuries among health care workers. Occup Med (Lond).

[4] Waters T, Collins J, Galinsky T, Caruso C (2006). NIOSH research efforts to prevent musculoskeletal disorders in the healthcare industry. Orthop Nurs.

[5] Hansen MC, Aagaard T, Christensen HW, Hartvigsen J (2018). Work-related acute physical injuries, chronic overuse complaints, and the psychosocial work environment in Danish primary care chiropractic practice - a cross-sectional study. Chiropr Man Therap..

[6] Mior SA, Diakow PR (1987). Prevalence of back pain in chiropractors. J Manip Physiol Ther.

[7] Lamprecht A, Padayachy K (2019). The epidemiology of work-related musculoskeletal injuries among chiropractors in the eThekwini municipality. Chiropr Man Therap.

[8] Rupert RL, Ebete KO (2004). Epidemiology of occupational injuries in chiropractic practice. J Chiropr Educ.

[9] Holm SM, Rose KA (2006). Work-related injuries of doctors of chiropractic in the United States. J Manip Physiol Ther.

[10] Dillman DA, Smyth JD, Christian LM (2014). Internet, phone, mail, and mixed-mode surveys: the tailored design method: John Wiley & Sons.

[11] Kuorinka I, Jonsson B, Kilbom A, Vinterberg H, Biering-Sørensen F, Andersson G (1987). Standardised Nordic questionnaires for the analysis of musculoskeletal symptoms. Appl Ergon.

[12] Bengtsson M (2016). How to plan and perform a qualitative study using content analysis. NursingPlus Open.

[13] Letvak SA, Ruhm CJ, Gupta SN (2012). Nurses' presenteeism and its effects on self-reported quality of care and costs. Am J Nurs.

