# Supplementary material for: Reported 1-year prevalence of occupational musculoskeletal disorders in Ontario chiropractors
Source: Chiropr Man Therap. 2020 Oct 23;28:55. doi: 10.1186/s12998-020-00345-2 (PMC7583170; doi:10.1186/s12998-020-00345-2)
Supplement: Supplementary file 1 — Survey of MSDs in Ontario chiropractors. Survey instrument used for online implementation. (PDF 56 kb) [file 12998_2020_345_MOESM1_ESM.pdf]

Section 1 – Demographics

Personal

1.1 Sex: ____ Male ____ Female

1.2 Age: __________ years

1.3 Height: __________ feet __________ inches

1.4 Weight: __________ pounds

1.5 From which Chiropractic College did you graduate? (drop down list of chiropractic colleges/programs in North America)

1.6 Do you have a practice specialization? (check all that apply)

____ Imaging ____ Clinical Sciences ____ Rehabilitation Sciences

____ Sport Sciences ____ Orthopedics

___ Other (please specify) _______________________

1.7 How many years have you been in practice? __________ years

Practice

1.8 What are the first three characters of the postal code for your practice location? __________

1.9 How many days do you typically work per week? __________ days

1.10 How many hours of patient contact do you have per week? __________ hours

1.11 How many hours of administrative work do you complete per week? __________ hours

1.12 Which chiropractic techniques do you use on a daily basis? (check all that apply)

____ Activator Methods ____ Applied Kinesiology ____ Cranial

____ Diversified ____ Flexion-Distraction ____ Gonstead

____ Logan Basic ____ Nimmo/Receptor Tonus ____ Palmer Upper Cervical/HIO

____ SOT ____Thompson

____ Other (please specify) _______________________

1.13 Which chiropractic technique do you use most frequently? (check only one)

____ Activator Methods ____ Applied Kinesiology ____ Cranial

____ Diversified ____ Flexion-Distraction ____ Gonstead

____ Logan Basic ____ Nimmo/Receptor Tonus ____ Palmer Upper Cervical/HIO

____ SOT ____Thompson

____ Other (please specify) ________________________

1.14 Which adjunct therapies do you use on a regular basis? (check all that apply)

____ Traction ____ Massage ____ Mobilization

____ Exercise ____ Acupuncture ____ Ice/Heat Packs

____ Ultrasound ____ Laser ____ Shockwave

____ Interferential Current (IFC)

____ Other (please specify) ________________________

1.15 Do you use a stationary or high-low table?

____ Stationary ____ High-low ____ Both

1.16 What is the typical height of your treatment table(s)?

__________ feet __________ inches

Section 2 – Musculoskeletal Disorder History

The next few questions will explore any musculoskeletal pain you have experienced in the past year, regardless of whether that pain is related to an injury sustained longer than one year ago

2.1 Have you at any time during the last 12-months experienced musculoskeletal trouble (ache, pain, discomfort), either work-related or not work-related, in any area of your body?

____ YES ____ NO

2.2 Would you say that your general health is:

____ 1 – Excellent ____ 2 – Very good ____ 3 – Good ____ 4 – Fair ____ 5 – Poor

If NO, then skip to end of questionnaire. If YES, then continue with subsequent questions.

2.3 How likely do you expect to completely recover from these musculoskeletal troubles?

__ 0 – Not likely __ 1 __ 2 __ 3 __ 4 __ 5 __ 6 __ 7 __ 8 __ 9 __ 10 – Very likely

2.4 Was your musculoskeletal trouble experienced in the last 12-months related to your work as a chiropractor?

____ YES ____ NO

If NO, then skip to Section 3 of the questionnaire. If YES, then continue with subsequent questions.

2.5 Have you at any time during the last 12-months had musculoskeletal trouble (ache, pain, discomfort) in your:

2.5.1 Neck? ____ YES ____ NO

2.5.2 Shoulders? ____ YES ____ NO

2.5.3 Elbows? ____ YES ____ NO

2.5.4 Wrists/Hands? ____ YES ____ NO

2.5.5 Upper Back? ____ YES ____ NO

2.5.6 Lower Back? ____ YES ____ NO

2.5.7 Hips/Thighs? ____ YES ____ NO

2.5.8 Knees? ____ YES ____ NO

2.5.9 Ankles/Feet? ____ YES ____ NO

If NO to all from 2.5.1 to 2.5.9, then skip to end of questionnaire. If YES to any of 2.5.1 to 2.5.9, then continue with subsequent questions for specific parts of the body.

Use the following set of questions if YES to any of 2.5.1 (Neck), 2.5.5 (Upper Back), or 2.5.6 (Lower Back). Here the X refers to a specific part of the body (e.g. X = 1 for Neck).

2.5.X.1 What activity were you doing when you first noticed the musculoskeletal troubles in your <BODY PART> in the past 12-months? (check only one)

____ Diagnostic procedure

____ Slipping, tripping, or falling

____ Applying modality

____ Maintaining prolonged position

____ Lifting

____ Demonstrating exercise

____ Positioning patient for manipulation/adjustment

____ Performing manipulation/adjustment

____ Do not remember

____ Other (please specify) ________________________

If either "Positioning patient for manipulation/adjustment" or "Performing manipulation/adjustment" are selected, then continue with question 2.5.X.1.1. Otherwise, continue with 2.5.X.2.

2.5.X.1.1 What part of the patient's body were you manipulating/adjusting?

____

2.5.X.1.2 What specific manipulative/adjustment technique were you using?

____

2.5.X.1.3 What position was the patient in?

____ Side-lying ____ Prone ____ Supine ____ Seated ____ Standing

2.5.X.1.4 What was the approximate height of the table?

__________ inches

2.5.X.2 Was the musculoskeletal trouble you experienced in your <BODY PART> in the past 12-months a first occurrence?

____ YES ____ NO

2.5.X.3 Have you at any time during the last 12-months changed jobs or duties because of <BODY PART> trouble?

____ YES ____ NO

2.5.X.4 What is the total length of time that you have had <BODY PART> trouble during the last 12-months?

____ 0 days ____ 1-7 days ____ 8-30 days ____ More than 30 days but not every day

____ Every day

If 0 days to 2.5.X.4, then skip to next reported body part with musculoskeletal trouble in the past 12-months. If no other reported body parts, then skip to Section 3. If anything other than 0 days reported for 2.5.X.4, then continue with 2.5.X.5.

2.5.X.5 Has <BODY PART> trouble caused you to reduce your **work activity** during the last 12-months?

____ No ____ Monthly ____ Weekly ____ Daily

2.5.X.6 Has <BODY PART> trouble caused you to reduce your **leisure activity** during the last 12-months?

____ No ____ Monthly ____ Weekly ____ Daily

2.5.X.7 What is the total length of time that <BODY PART> trouble has prevented you from doing your normal work?

____ 0 days ____ 1-7 days ____ 8-30 days ____ More than 30 days

2.5.X.8 Have you sought medical or paramedical help for your <BODY PART> trouble in the past 12-months?

____ YES ____ NO

Use the following set of questions if YES to any of 2.5.2 (Shoulders), 2.5.3 (Elbows), 2.5.4 (Wrists/Hands), 2.5.7 (Hips/Thighs), 2.5.8 (Knees), or 2.5.9 (Ankles/Feet). Here the X refers to a specific part of the body (e.g. X = 2 for Shoulders).

2.5.X.1 What side of the body is your <BODY PART> trouble located on?

____ LEFT ____ RIGHT ____ BOTH

2.5.X.2 What activity were you doing when you first noticed the musculoskeletal troubles in your <BODY PART> in the past 12-months? (check only one)

____ Diagnostic procedure

____ Applying modality

____ Lifting

____ Positioning patient for manipulation/adjustment

____ Performing manipulation/adjustment

____ Other (please specify) ________________________

____ Slipping, tripping, or falling

____ Maintaining prolonged position

____ Demonstrating exercise

____ Do not remember

If either "Positioning patient for manipulation/adjustment" or "Performing manipulation/adjustment" are selected, then continue with question 2.5.X.2.1. Otherwise, continue with 2.5.X.3.

2.5.X.2.1 What part of the patient's body were you manipulating/adjusting?

____

2.5.X.2.2 What specific manipulative/adjustment technique were you using?

____

2.5.X.2.3 What position was the patient in?

____ Side-lying ____ Prone ____ Supine ____ Seated ____ Standing

2.5.X.2.4 What was the approximate height of the table?

__________ inches

2.5.X.3 Was the musculoskeletal trouble you experienced in your <BODY PART> in the past 12-months a first occurrence?

____ YES ____ NO

2.5.X.4 Have you at any time during the last 12-months changed jobs or duties because of <BODY PART> trouble?

____ YES ____ NO

2.5.X.5 What is the total length of time that you have had <BODY PART> trouble during the last 12-months?

____ 0 days ____ 1-7 days ____ 8-30 days ____ More than 30 days but not every day

____ Every day

If 0 days to 2.5.X.5, then skip to next reported body part with musculoskeletal trouble in the past 12-months. If no other reported body parts, then skip to Section 3. If anything other than 0 days reported for 2.5.X.5, then continue with 2.5.X.6.

2.5.X.6 Has <BODY PART> trouble caused you to reduce your **work activity** during the last 12-months?

____ YES ____ NO

2.5.X.7 Has <BODY PART> trouble caused you to reduce your **leisure activity** during the last 12-months?

____ YES ____ NO

2.5.X.8 What is the total length of time that <BODY PART> trouble has prevented you from doing your normal work?

____ 0 days ____ 1-7 days ____ 8-30 days ____ More than 30 days

2.5.X.9 Have you sought medical or paramedical help for your <BODY PART> trouble in the past 12-months?

____ YES ____ NO

Section 3 – Work Modifications Related to Musculoskeletal Disorder History

3.1 Did you make any changes to your practice (e.g. volume, hours worked, patient type) as a result of your musculoskeletal trouble(s)?

____ YES ____ NO

If YES to 3.1, then ask for a detailed account of specific changes that were made.

3.1.1 Please provide a detailed account of specific changes to your practice (e.g. volume, hours worked, patient type) as a result of your musculoskeletal trouble(s)?

____

____

____

____

____

3.2 Did you make any changes to techniques used and/or technique parameters (e.g. patient positioning, amount of force, your posture) as a result of your musculoskeletal trouble(s)?

____ YES ____ NO

If YES to 3.2, then ask for a detailed account of specific changes that were made.

3.2.1 Please provide a detailed account of specific changes to techniques used and/or technique parameters (e.g. patient positioning, amount of force, your posture) as a result of your musculoskeletal trouble(s).

____

____

____

____

____
